# Residues 140–142, 199–200, 222–223, and 262 in the Surface Glycoprotein of Subgroup A Avian Leukosis Virus Are the Key Sites Determining Tva Receptor Binding Affinity and Infectivity

**DOI:** 10.3389/fmicb.2022.868377

**Published:** 2022-04-27

**Authors:** Jinqun Li, Jian Chen, Xinyi Dong, Canxin Liang, Yanyan Guo, Xiang Chen, Mengyu Huang, Ming Liao, Weisheng Cao

**Affiliations:** ^1^College of Veterinary Medicine, South China Agricultural University, Guangzhou, China; ^2^Key Laboratory of Zoonosis Prevention and Control of Guangdong Province, Guangzhou, China; ^3^National and Regional Joint Engineering Laboratory for Medicament of Zoonosis Prevention and Control, South China Agricultural University, Guangzhou, China; ^4^Key Laboratory of Veterinary Vaccine Innovation of the Ministry of Agriculture, Guangzhou, China; ^5^Key Laboratory of Zoonosis of Ministry of Agriculture and Rural Affairs, Guangzhou, China

**Keywords:** avian leukosis viruses, Tva, surface glycoprotein, binding affinity, infectivity

## Abstract

Subgroup A avian leukosis virus (ALV-A) invades cells through *gp85*-encoded surface glycoprotein (SU) *via* specifically recognizing the cellular receptor Tva. To identify the key residues of ALV-A SU that determine the Tva binding affinity and infectivity in DF-1 cells, a strategy of substituting corresponding residues of SU between ALV-A RSA and ALV-E ev-1 (using Tvb as the receptor) was adopted. A series of chimeric soluble gp85 proteins were expressed for co-immunoprecipitation (co-IP) analysis and blocking analysis of viral entry, and various recombinant viruses based on replication-competent avian retrovirus vectors containing Bryan polymerase (RCASBP) were constructed for transfection into DF-1 cells and measurement of the percentage of GFP-positive cells. The results revealed that the substitution of residues V138, W140, Y141, L142, S145, and L154 of host range region 1 (hr1), residues V199, G200, Q202, R222, and R223 of host range region 2 (hr2), and residue G262 of variable region 3 (vr3) reduced the viral infectivity and Tva binding affinity, which was similar to the effects of the −139S, −151N, −155PWVNPF, −201NFD, Δ214–215, and −266S mutations. Our study indicated that hr1 and hr2 contain the principal receptor interaction determinants, with new identified-vr3 also playing a key role in the receptor binding affinity of ALV-A.

## Introduction

Avian leukosis virus (ALV), a member of the genus *Alpharetrovirus* and the family *Retroviridae*, has been classified into 11 subgroups from A to K based on the host range, antibody neutralization, and interference in superinfection experiments (Chesters et al., 2002; Zhang et al., 2014; Prikryl et al., 2019). Subgroup A of ALV (ALV-A), an exogenous ALV subgroup associated with neoplastic and immunosuppressive diseases, could cause serious economic losses to the poultry industry, as there is no effective vaccine or drug to control infection (Wang et al., 2020; Lipsick, 2021).

Like most retroviruses, ALV initially synthesizes its envelope glycoprotein (Env) as a precursor that is subsequently processed into two subunits: *gp85*-encoded surface glycoprotein (SU), which contains the main domains that interact with the host receptor, and transmembrane glycoprotein (TM), which anchors SU on the membrane with a stable covalent disulfide bond (Smith and Cunningham, 2007; Li et al., 2020; Deng et al., 2021). The SU glycoproteins of ALV-A through ALV-E are highly conserved except for two host range regions (hr1 and hr2) and three variable regions (vr1, vr2, and vr3) (Federspiel, 2019). Previous studies have revealed that the hr1 and hr2 domains contain the principal determinants of receptor interaction, while the vr3 domain contributes to the specificity of receptor recognition for initiating effective infection but not to receptor binding affinity (Holmen et al., 2001; Melder et al., 2003; Federspiel, 2019; Munguia and Federspiel, 2019). The variable regions of vr1 and vr2 did not appear to be required for binding affinity or receptor specificity (Dorner et al., 1985; Melder et al., 2003).

For ALVs to invade cells, their envelope proteins must primarily bind to cell surface receptor proteins (Federspiel, 2019). Unlike human immunodeficiency virus (HIV), which needs dual coreceptors to invade cells (Weichseldorfer et al., 2022), ALVs, a group of simple retroviruses similar to mouse leukemia virus (MLV; Bova et al., 1988), require only a single functional receptor to infect target cells. Members of different families of proteins have been identified as receptors of ALV: Tva for ALV-A/K (Prikryl et al., 2019), Tvb for ALV-B/D/E (Adkins et al., 2000; Brojatsch et al., 2000), Tvc for ALV-C (Elleder et al., 2005), and chicken Na^+^/H^+^ exchanger type 1 for ALV-J (Chai and Bates, 2006). Some studies on the functional domain of ALV receptor have confirmed that a few specific amino acid residues play a key role in binding to ALV Env proteins and mediating viral infection (Rong and Bates, 1995; Klucking and Young, 2004; Guan et al., 2018; Kheimar et al., 2021). However, under the selective pressure of entry competitors, ALVs have the ability to evolve the structure of their Env proteins to use different cellular proteins as receptors (Munguia and Federspiel, 2019).

The SU glycoproteins of ALV-A RSA (GenBank: M37980.1) and the endogenous virus ALV-E ev-1 (GenBank: AY013303.1) used in this study show high homology, and the regions with differences are mainly located in hr1, hr2, and vr3. However, different receptors are involved in the invasion and sensitivity of DF-1 cells, a permanent, non-transformed cell line derived from Line 0 chicken embryo fibroblasts, which is insensitive to ALV-E (Federspiel et al., 1991). Therefore, the differences between the binding sites of ALV-A and ALV-E determine the mechanism of cell invasion. In other words, hr1, hr2, and vr3 may be the key regions determining the Tva receptor binding affinity and infectivity in DF-1 cells.

To verify this conjecture, a series of chimeric gp85 proteins and recombinant viruses were evaluated by replacing the residues corresponding to ALV-E ev-1 for Tva binding and viral infectivity. Our results indicated that hr1 and hr2 contain the principal binding domains between SU and the Tva receptor, with vr3 playing a key role in the receptor binding affinity of ALV-A.

## Materials and Methods

### Cell Cultures and Antibodies

DF-1 cells (from ATCC, kept in our lab) were grown in Dulbecco’s Modified Eagle’s Medium (DMEM; Gibco, Carlsbad, CA, United States) supplemented with 10% fetal bovine serum (FBS; Gibco, Australia), 100 units/ml of penicillin, and 100 mg/ml of streptomycin (Gibco, Carlsbad, CA, United States) in the presence of 5% CO_2_ at 39°C. 293T cells were cultured in DMEM supplemented with 10% FBS at 37°C in a 5% CO_2_ atmosphere. The mouse anti-HA tag antibody was purchased from Thermo Fisher Scientific Inc. (Rockford, IL, United States), whereas the mouse anti-flag M2 tag antibody and the rabbit anti-GAPDH antibodies were purchased from Sigma (Sigma-Aldrich, St. Louis, MO, United States). IRDye 680RD goat anti-mouse IgG (H + L) antibody was purchased from LI-COR Biosciences (Lincoln, NE, United States).

### Construction of Recombinant RCASBP Retroviral Vector With ALV-A RSA and ALV-E ev-1

The construction of the RCASBP (A)-EGFP retroviral vector [the ALV-based replication-competent RCASBP vector with the ALV-A RSA *env* gene and the enhanced green fluorescent protein (EGFP) gene] and the RCASBP (E)-EGFP retroviral vector has been described previously (Chen et al., 2020). Similar to the method of constructing RCASBP (K/E)-EGFP described previously (Chen et al., 2020), a series of fragments containing the 3′ end of *pol*, mutant SU regions, and complete TM regions were amplified by overlapping PCR with corresponding primers (Supplementary Table 1) and cloned into the unique *KpnI* and *StuI* sites of RCASBP (A)-EGFP to construct various recombinant RCASBP (A/E)-EGFP vectors.

### Construction of Plasmids Expressing the Tva Receptor and Various Chimeric Soluble gp85 Proteins

The eukaryotic plasmid pCAGGS-Tva-HA-Fc, encoding chicken Tva, HA tag, and the human IgG-Fc fragment, which specifically binds to the protein A/G (Lexington, MA, United States), has been described previously (Chen et al., 2020). To express gp85 protein in a soluble form and facilitate identification, a signal peptide designated “s” and 3 × flag tags were fused to its N-terminus and C-terminus, respectively. The *s-gp85-flag* sequence was subsequently cloned into the *EcoRI* and *BglII* sites of pCAGGS to construct a eukaryotic plasmid (pCAGGS-s-RSA-gp85-flag). Similar to the method of constructing RCASBP (A/E)-EGFP, the residues of RSA SU were replaced with the corresponding ev-1 residues by overlapping PCR with corresponding primers (Supplementary Table 2), and various recombinant pCAGGS-s-gp85-flag plasmids encoding chimeric soluble gp85 proteins were constructed by homologous recombination.

### Fluorescence-Activated Cell Sorting Analysis of Recombinant Virus Infecting DF-1 Cells

Virus propagation was initiated by the transfection of plasmid DNA that contained the retroviral vector in proviral form. DF-1 cells in 6-well plates were transfected with 1 μg of RCASBP (A/E)-EGFP vector using Lipofectamine 3000 reagent (Shanghai, China) and passaged at 48 h posttransfection. The cells were then visualized under a Leica DMI 4000B fluorescence microscope (Leica, Wetzlar, Germany) and collected to determine the percentage of GFP-positive cells by FACS using an LSRII analyzer (Becton, Dickinson and Company, United States) at 5 or 7 days posttransfection.

### Blocking Analysis of gp85 Protein Binding to Receptor

Using PolyJet DNA transfection reagent (Rockville, MD, United States), 4 μg optimal transfection amount (Supplementary Figure 1) of the respective recombinant pCAGGS-s-gp85-flag plasmid was transfected into 293T cells with 95% confluence in a 60 mm culture dish. At 8 h posttransfection, the cell supernatant with transfection mixtures was replaced by 3 mL of fresh DMEM with 1% FBS. The supernatant was subsequently collected at 48 h posttransfection as the source of recombinant gp85 proteins. DF-1 cells with 75% confluence in 24-well plates were washed with phosphate-buffered saline (PBS) and incubated with chimeric gp85 proteins for 1 h at 4°C. After discarding the supernatant, DF-1 cells were incubated with 0.1 multiplicity of infection (MOI) ALV-A RSA obtained by RCASBP (A)-EGFP transfection for 2 h at 39°C. The cells were then washed 5 times with PBS and cultured in 1% FBS DMEM for 5 days to measure the percentage of GFP-positive cells by FACS. The higher the binding affinity of the gp85 protein to the Tva receptor was, the lower the percentage of GFP-positive cells.

### Co-immunoprecipitation and Pull-Down Assay

To express chimeric soluble gp85 proteins, 4 μg of pCAGGS-s-gp85-flag plasmid was transfected into 293T cells in a 60 mm culture dish using PolyJet DNA transfection reagent. At 48 h posttransfection, the culture supernatant was collected and the cells were lysed with NP-40 buffer (Beyotime Biotechnology, Shanghai, China) for the next experiment. Cleared supernatant harvested from 293T cells transfected with pCAGGS-Tva-HA-Fc was concentrated to 1/10 volume through a 10 kDa molecular weight cutoff ultrafiltration spin columns (Merck Millipore, Darmstadt, Germany) and the Tva proteins were purified using 60 μL of Protein A/G agarose for 2 h at 4°C with gentle agitation. After washing 5 times with ice-cold PBS, the Protein A/G agarose was incubated with recombinant soluble gp85 proteins for 5 h at 4°C. After washing again with ice-cold PBS, the bound proteins were denatured by heating for 5 min at 100°C with SDS–PAGE sample loading buffer.

### Western Blotting

The denatured protein was separated by 10% SDS–PAGE (Beyotime Biotechnology, Shanghai, China) and transferred to nitrocellulose membranes (Merck Millipore, Darmstadt, Germany). After blocking with 5% non-fat powdered milk for 1 h at room temperature and washing 3 times with TBST (Tris-buffered saline containing 0.1% Tween 20), the membranes were incubated with anti-FLAG or anti-HA monoclonal antibody at 4°C overnight. After washing again with TBST, the membranes were incubated with IRDye 680RD goat anti-mouse IgG (H + L) antibody for 1 h at room temperature. Finally, an Odyssey Infrared Imaging System (LI-COR Biosciences, Lincoln, NE, United States) was used to scan the membrane spots.

### Statistical Analysis

Data are shown as the means ± standard deviations in triplicate from a representative experiment and were analyzed by Student’s *t*-test using GraphPad Prism 7. A *P* value of <0.05 was considered significant. *, ^**^, ^***^, and ^****^ indicate *P* values less than 0.05, 0.01, 0.001, and 0.0001, respectively. These experiments were performed independently at least three times with similar results.

## Results

### Low Homology Between ALV-A and ALV-E in the hr1, hr2, and vr3 Regions

**FIGURE 1:**
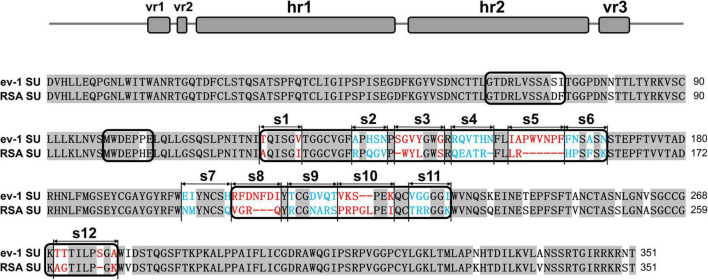
Schematic representations of the replacement fragments and comparison between the amino acid sequences of RSA and ev-1 SU. The amino acid sequences were aligned by using the ClustalW method in the MegAlign program (DNASTAR, Madison, WI, United States). The residues from hr1 to hr2 and the vr3 region were divided into 12 segments (designated s1–s12). ev-1, Subgroup E of avian leukosis virus strain ev-1, GenBank AY013303.1; RSA, Subgroup A of avian leukosis virus strain RSA, GenBank M37980.1. Red and blue letters indicated substitutions of residues in different fragments.

The SU glycoproteins of ALV-A RSA and ALV-E ev-1 were highly conserved, exhibiting 83.5% homology at the amino acid level according to the ClustalW method in the MegAlign program, with the greatest degree of variability in the hr1, hr2, and vr3 regions. The vr1 and vr2 regions were relatively stable, with changes of only two amino acids and one amino acid, respectively. The residues from hr1 to hr2 and the vr3 region were divided into 12 segments (designated s1–s12) for subsequent research (Figure 1).

### Substitution of Residues s2–s6 of ALV-A With ALV-E Reduced Tva Binding Affinity and Infectivity in DF-1 Cells

**FIGURE 2:**
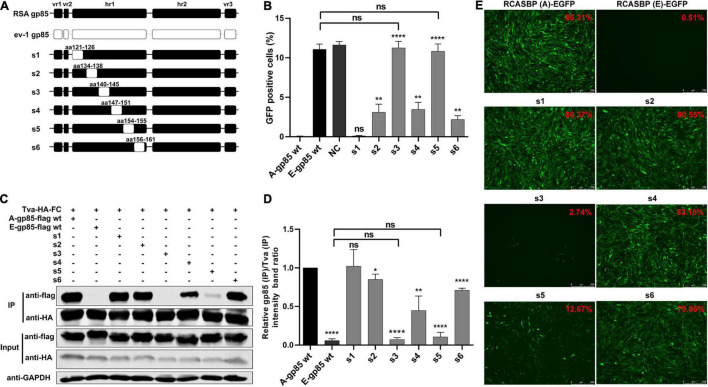
Substitution of residues s1–s6 of ALV-A with ALV-E reduced the Tva binding affinity and infectivity of DF-1 cells. **(A)** Schematic representations of the replacement fragments s1–s6 in hr1. **(B)** DF-1 cells were incubated with chimeric gp85 protein s1–s6 and subsequently infected with RCASBP (A)-EGFP supernatants. The percentage of GFP-positive cells was measured by FACS for blocking analysis of the binding of chimeric gp85 protein to Tva. **(C)** The interaction of chimeric gp85 proteins s1–s6 with Tva-HA-Fc. **(D)** The gray scale value of the protein band was quantified using Image Studio Lite Version 5.2, and gp85 (IP)/Tva (IP) was calculated. **(E)** DF-1 cells transfected with recombinant RCASBP (A/E)-s1-s6-EGFP vectors were visualized under a fluorescence microscope, and the percentage of GFP-positive cells (indicated with red color letters) was detected by FACS 7 days posttransfection. *P < 0.05, **P < 0.01, ****P < 0.0001.

To determine the role of hr1 in the infection and receptor binding of ALV-A, hr1 was divided into six segments and replaced with the corresponding regions of ev-1 to construct a series of chimeric gp85 proteins s1–s6 and recombinant virus vectors RCASBP (A/E)-s1-s6-EGFP (Figure 2A). DF-1 cells were separately incubated with s1–s6 chimeric gp85 protein and subsequently infected with RCASBP (A)-EGFP recombinant virus. As expected, the entry of RCASBP (A)-EGFP was blocked by wild-type (wt) A-gp85, and the GFP-positive signal was not present at experimentally detectable levels, in contrast to E-gp85 wt (Figure 2B). The chimeric gp85 proteins s1 completely blocked viral entry, whereas s2–s6 significantly reduced the blocking effect, showing higher percentages of GFP-positive cells than A-gp85 wt (*p* < 0.01). Moreover, the substitution of residues s3 and s5 showed the maximum reduction in the blocking effect (*p* < 0.0001), which was not statistically different from the result of E-gp85 wt (Figure 2B).

Since the blocking effect of gp85 proteins depends on competitive binding to the Tva receptor on the surface of DF-1 cells, the above results suggested that the substitution of residues s2–s6 reduced the binding affinity to the Tva receptor. For further verification, the chimeric soluble gp85 proteins s1–s6 were used for co-IP (Figure 2C). The gray values of s2, s3, s4, s5, and s6 were significantly lower than that of A-gp85 wt (*P* < 0.05). In particular, the substitution of residue s3 eliminated the interaction with Tva, with almost no gray signal detected (Figures 2C,D).

Furthermore, DF-1 cells separately transfected with recombinant RCASBP (A/E)-s1-s6-EGFP vectors were visualized under a fluorescence microscope and collected to determine the percentage of GFP-positive cells by FACS. As expected, the substitution of residues s2–s6 reduced the percentage of GFP-positive cells. Moreover, the substitution of s3 almost eliminated the ability to infect DF-1 cells, exhibiting only 2.74% GFP-positive cells (Figure 2E).

### Substitution of Residues s7–s12 of ALV-A With ALV-E Reduced the Tva Binding Affinity and Infectivity in DF-1 Cells

**FIGURE 3:**
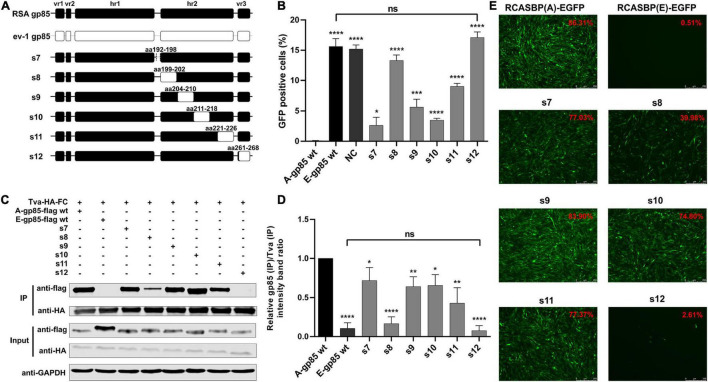
Substitution of residues s7–s12 of ALV-A with ALV-E reduced the Tva binding affinity and infectivity in DF-1 cells. **(A)** Schematic representations of the replacement fragments s7–s12. **(B)** DF-1 cells were incubated with chimeric gp85 protein s7–s12 and subsequently infected with RCASBP (A)-EGFP supernatants. The percentage of GFP-positive cells was measured by FACS for blocking analysis of the binding of chimeric gp85 proteins to Tva. **(C)** The interaction of chimeric gp85 protein s1–s6 with Tva-HA-Fc. **(D)** The gray scale value level of gp85 (IP)/Tva (IP) was calculated. **(E)** DF-1 cells transfected with recombinant RCASBP (A/E)-s1-s6-EGFP vectors were visualized under a fluorescence microscope and the percentage of GFP-positive cells (indicated with red color letters) was detected by FACS 7 days posttransfection. *P < 0.05, **P < 0.01, ***P < 0.001, ****P < 0.0001.

To further investigate the effects of the region between hr1 and hr2, hr2, and vr3 on the receptor binding affinity and infectivity in DF-1 cells, a series of chimeric gp85 proteins s7–s12 were expressed for co-IP and blocking analysis and various recombinant virus vectors RCASBP (A/E)-s7-s12-EGFP were constructed for viral entry assays (Figure 3A). The blocking analysis showed that the percentages of GFP-positive cells of chimeric proteins s7–s12 were significantly higher than those of A-gp85 wt (*p* < 0.05). In particular, the substitution of the vr3 region (s12) exhibited no blocking effect on viral entry (*p* < 0.0001), similar to E-gp85 wt (Figure 3B). The results of protein interactions revealed that chimeric proteins s7–s12 all had significantly reduced Tva binding affinity (*p* < 0.05). In particular, the substitution of vr3 eliminated the interaction with Tva (Figures 3C,D). To further verify the influence of these residues on infectivity, wild-type and mutant virus vectors RCASBP (A/E)-s7-s12-EGFP were transfected into DF-1 cells, and the percentage of GFP-positive cells was monitored. As expected, all six mutant ALVs had a replication disadvantage over wild-type ALV-A in DF-1 cells, in which the substitution of the vr3 region basically eliminated the ability to infect DF-1 cells, resulting in only 2.61% GFP-positive cells (Figure 3E).

### Identification of the Key Residues in the s3, s5, s8, and vr3 Regions for ALV-A Binding Receptor and Invading Cells

**FIGURE 4:**
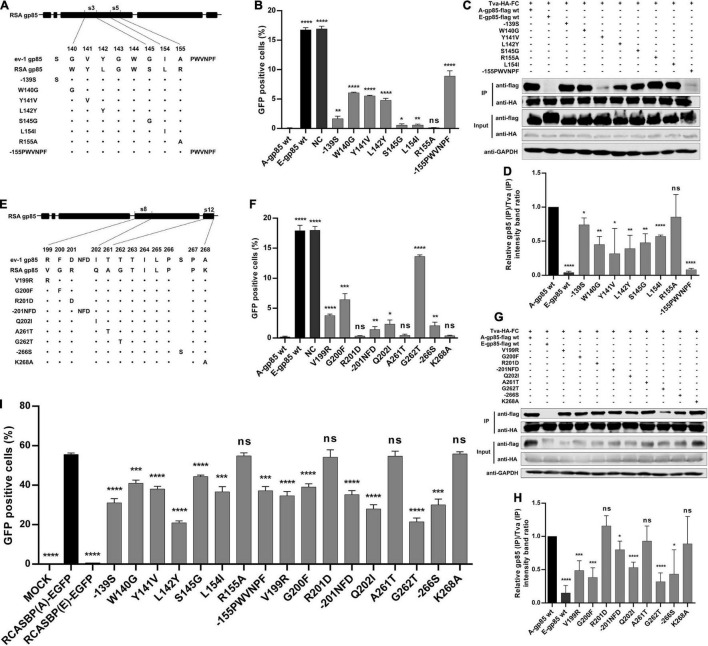
Identification of the key residues in the s3, s5, s8, and vr3 regions for the ALV-A binding receptor and invading cells. **(A,E)** Schematic representation of amino acid substitutions in s3 and s5 or s8 and s12. **(B,F)** DF-1 cells were incubated with mutant gp85 proteins with amino acid substitutions in s3 and s5 or s8 and s12 and subsequently infected with RCASBP (A)-EGFP. The percentage of GFP-positive cells was measured by FACS for blocking analysis. **(C,G)** The interaction of the mutant gp85 protein with amino acid substitutions in s3 and s5 or s8 and s12 with Tva-HA-Fc. **(D,H)** The gray scale value level of gp85 (IP)/Tva (IP) was calculated. **(I)** DF-1 cells transfected with recombinant RCASBP (A/E)-EGFP vectors with mutations V138N, −151N, Δ214–215, R222G, or R223G were detected for the percentage of GFP-positive cells by FACS 5 days posttransfection. *P < 0.05, **P < 0.01, ***P < 0.001, ****P < 0.0001.

In the above experiment, substitution of the s3, s5, s8, and s12 regions showed the maximum reduction in receptor binding affinity and infectivity in DF-1 cells. To identify which amino acid residues in s3 and s5 of hr1 determine the interaction of gp85 and Tva, various mutant gp85 proteins with amino acid substitutions in s3 and s5 were expressed for competitive blocking analysis and co-IP assay (Figure 4A). While R155A did not, the mutant gp85 proteins −139S, W140G, Y141V, L142Y, S145G, L154I, and −155PWVNPF all reduced but not eliminated the blocking effect of viral entry, as their percentages of GFP-positive cells were significantly higher than that of A-gp85 wt (*p* < 0.05) but lower than that of E-gp85 wt, of which W140G, Y141V, L142Y, and −155PWVNPF had the maximum reduction (*p* < 0.0001, Figure 4B). As expected, the co-IP assay showed a similar result: the mutant gp85 protein with amino acid substitutions in s3 and s5 all significantly reduced the binding affinity with Tva except R155A (*p* < 0.05, Figures 4C,D).

Similarly, a series of recombinant gp85 proteins targeting single residue mutations in S8 of hr2 and s12 of vr3 were also constructed for blocking analysis and co-IP assay (Figure 4E). The percentage of GFP-positive cells suggested that the V199R, G200F, −201NFD, Q202I, G262T, and −266S mutations significantly inhibited interaction with the Tva receptor (*p* < 0.05). In particular, the G262T mutation in vr3 had the maximum reduction in Tva binding affinity (Figure 4F). The co-IP assay also showed a similar result (Figures 4G,H).

For further verification, the corresponding recombinant RCASBP (A/E)-EGFP vectors with amino acid substitutions in s3, s5, s8, and s12 were transfected into DF-1 cells, and the infection ability was detected by FACS (Figure 4I). As expected, the percentages of GFP-positive cells for the R155A, R201D, A261T, and K268A mutations were not statistically different from that of RCASBP (A)-EGFP, whereas the mutations −139S, W140G, Y141V, L142Y, S145G, L154I, −155PWVNPF, V199R, G200F, −201NFD, Q202I, G262T, and −266S significantly reduced the level of virus replication in DF-1 cells (*p* < 0.001, Figure 4I). These results suggested that the residues of W140, Y141N, L142, S145, and L154 in hr1; V199, G200, and Q202 in hr2; and G262 in vr3 play key roles in determining receptor binding affinity and infectivity of DF-1 cells.

### Identification of the Key Residues in the s2, s4, s6, s7, s9, and s11 Regions for ALV-A Binding Receptor and Invading Cells

**FIGURE 5:**
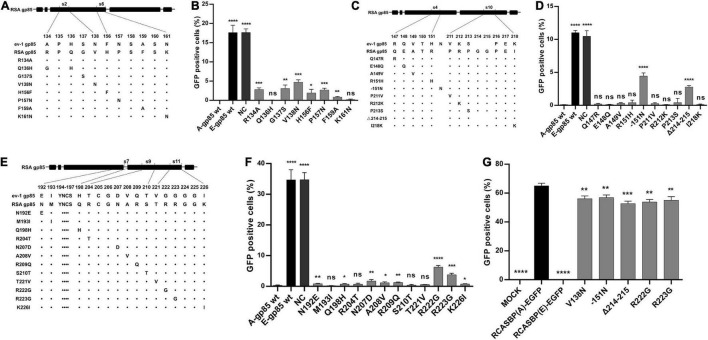
Identification of the key residues in the s2, s4, s6, s7, s9, s10, and s11 regions for the ALV-A binding receptor and invading cells. **(A,C,E)** Schematic representation of amino acid substitutions in s2 and s6, s4 and s10, or s7, s9, and s11. **(B,D,F)** DF-1 cells were incubated with mutant gp85 proteins with amino acid substitutions in s2 and s6 or s4 and s10 or s7, s9, and s12 and subsequently infected with RCASBP (A)-EGFP. The percentage of GFP-positive cells was measured by FACS for blocking analysis. **(G)** DF-1 cells transfected with recombinant RCASBP (A/E)-EGFP vectors with amino acid substitutions in s3, s5, s8, and s12 were detected for the percentage of GFP-positive cells by FACS 5 days posttransfection. *P < 0.05, **P < 0.01, ***P < 0.001, ****P < 0.0001.

In addition to s3, s5, s8, and s12, the substitution of residues s2, s4, s6, s7, s9, s10, or s11 of ALV-A RSA with ALV-E ev-1 also reduced the receptor binding affinity and infectivity in DF-1. To further determine which amino acid residue in these regions plays a key role, a series of mutant gp85 proteins with amino acid substitutions in s2, s4, s6, s7, s9, s10, and s11 were expressed and divided into three groups for the competitive blocking test (Figures 5A,C,E). The results showed that the blocking effect of the mutations R134A, G137S, V138N in s2, and H156F, P157N, F159A in s6 was statistically reduced (*p* < 0.05, Figure 5B), which was similar to the effect of the mutations −151N in s4 and Δ214–215 in s10 (*p* < 0.0001, Figure 5D), as well as N192E, Q198H in s7, N207D, A208V, R209Q in s9, and R222G, R223G, K226I in s11 (*p* < 0.05, Figure 5F). For further confirmation, five recombinant RCASBP (A/E)-EGFP vectors with the mutations V138N, −151N, P157N, Δ214–215, R222G, and R223G, which had the maximum reduction in receptor binding affinity above, were transfected into DF-1 cells, and the percentage of GFP-positive cells was detected by FACS. As expected, all five mutations inhibited virus replication in DF-1 cells (*p* < 0.001, Figure 5G).

### Back Mutation Restored the Interaction Between gp85 and Tva

**FIGURE 6:**
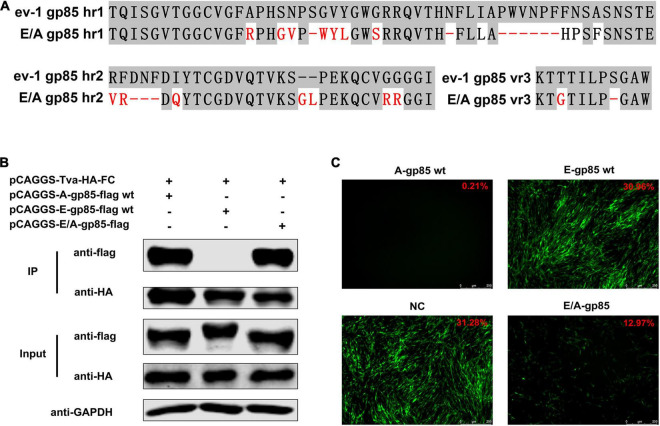
Back mutation restored the interaction between gp85 and Tva. **(A)** Schematic representation of back mutation. **(B)** The interaction of A-gp85-flag wt, E-gp85-flag wt, and back mutant protein E/A-gp85-flag with Tva-HA-Fc. **(C)** DF-1 cells were incubated with A-gp85 wt, E-gp85 wt, and back mutant E/A-gp85 protein and subsequently infected with RCASBP (A)-EGFP. The cells were visualized under a fluorescence microscope and the percentage of GFP-positive cells (indicated with red color letters) was detected by FACS for blocking analysis.

To verify the identified key amino acid residues determining the binding affinity of ALV-A SU to Tva, using ALV-E ev-1 SU as the skeleton, the gp85 protein was replaced with the corresponding regions of ALV-A RSA (A134R, G137S, N138V, S140-, G141W, V142Y, Y143L, G146S, N153-, I156L, Δ158–163, F164H, N165P, A167F, R207V, F208G, Δ209–211, I213Q, −224GL, G231R, G232R, T272G, and S277-) to construct a back mutant protein E/A-gp85 (Figure 6A). As expected, co-IP and pull-down assays showed that the back mutant E/A-gp85 protein could interact with Tva protein (Figure 6B). Moreover, the back mutation partially restored the blocking effect of RCASBP (A)-EGFP in the infection of DF-1 cells, exhibiting only 12.97% GFP-positive cells (Figure 6C), which was lower than that of E-gp85 wt (30.96%) but higher than that of A-gp85 wt (0.21%). Therefore, the corresponding residues R134, G137, V138, W140, Y141, L142, S145, L154, H156, P157, and F159 in hr1; V199, G200, Q202, G214, L215, R222, and R223 in hr2; and G262 in vr3 were the key sites for ALV-A SU binding to the Tva receptor.

## Discussion

Subgroup A-E avian leukosis viruses are a group of highly related retroviruses that evolved their env genes encoding viral envelope glycoproteins from a common ancestor to use very different members of the host protein families as receptors, enabling efficient viral entry (Federspiel, 2019). Among them, ALV-A shares Tva as a receptor with ALV-K (Prikryl et al., 2019), while ALV-E shares the tumor necrosis factor receptor encoded by three alleles (tvbs^1^, tvbs^3^, and tvb^st^) as a receptor with ALV-B/D (Adkins et al., 2000). The amino acid differences of Env between ALV-A and ALV-E are concentrated in three regions (hr1, hr2, and vr3) of the SU glycoprotein (Figure 1). Therefore, hr1, hr2, and vr3 may be the key regions determining Tva receptor binding affinity and infectivity in DF-1 cells, a permanent cell line that is sensitive to exogenous ALV but insensitive to ALV-E (Federspiel et al., 1991). To verify this hypothesis, using a strategy of substituting corresponding residues of SU between ALV-A RSA and ALV-E ev-1, a series of chimeric gp85 proteins were expressed for blocking analysis and co-IP assay, while various recombinant virus vectors based on RCASBP were transfected into DF-1 cells for infectivity analysis. The results revealed that the substitution of residues s2 (aa134–138) to s12 (aa261–268) all reduced the blocking effect against RCASBP (A)-EGFP, the binding affinity of Tva, and replication in DF-1 cells (Figures 2, 3), and residues 138, 140, 141, 142, 145, 154 in hr1, residues 199, 200, 222, 223 in hr2, and residue 262 in vr3 were the key sites determining receptor binding affinity and infectivity in DF-1 cells (Figures 4–6). In particular, the substitution of residues 140–142, 199–200, 222–223, and 262 of ALV-A with ALV-E exhibited no blocking effect on viral entry (Supplementary Figure 2). Our study indicates that hr1 and hr2 contain the principal receptor interaction determinants, with vr3 also playing a key role in the receptor binding affinity in ALV-A.

In previous studies on retroviruses, the domain of SU glycoprotein that binds to host cell receptors can be classified into two types: one is concentrated in the highly variable region in SU, as in mouse leukemia virus and ALV-A/B/C/D/E/K (Battini et al., 1995; Federspiel, 2019; Chen et al., 2020); the other is composed of a complex of discontinuous and multivariate segments of the SU protein, as in human immunodeficiency virus, equine infectious anemia virus, and ALV-J (Kwong et al., 1998; Sun et al., 2008; Zhang et al., 2020). Previous studies have determined that hr1 and hr2 are the principal binding domains between the viral glycoprotein trimer and the host protein receptor (Tsichlis et al., 1980; Federspiel, 2019). Consistently our research showed that the substitution of residues s2–s6 (aa134–161) in hr1 (Figure 2) and s8–s11 (aa192–216) in hr2 (Figure 3) of ALV-A all had a negative influence on the infectivity of DF-1 cells and the interaction between SU and the receptor. Specifically, residues R134, G137, V138, W140, Y141, L142, S145, L154, H156, P157, and F159 in hr1 and V199, G200, Q202, G214, L215, R222, and R223 in hr2 were the key sites for ALV-A SU binding to the Tva receptor (Figures 4, 5). Moreover, in our previous studies, we replaced the residues between hr1 and hr2 of ALV-K with those of ALV-E and found that the mutant SU retained its binding affinity to Tva, but the infectivity of the recombinant virus was almost completely negated (Chen et al., 2020). However, this study revealed that the domain between hr1 and hr2 of ALV-A could affect not only the infectivity in DF-1 cells but also the binding affinity of SU and Tva to a certain extent (Figure 3), although it did not appear to be required for restoring the interaction between the back mutant SU and the Tva receptor (Figure 6).

It is worth noting that although previous studies have revealed that the vr3 domain contributes to the specificity of receptor recognition for initiating effective infection but not to receptor binding affinity (Federspiel, 2019), our results indicated that the vr3 region plays an essential role in the direct binding affinity between ALV-A SU and Tva receptor. After we replaced the vr3 domain of ALV-A with ALV-E, the recombinant virus hardly infected DF-1 cells and the recombinant gp85 protein appeared to lose its binding affinity to Tva (Figures 3B–D). Further single amino acid substitution demonstrated that residue G262 is essential for the binding of SU to Tva, mutation of G262 significantly reduced the blocking effect of viral entry, the Tva receptor binding affinity, and infectivity of the recombinant virus (Figures 4F–I).

The cellular receptor of ALV-A, Tva, utilizes a 40-residue, acidic domain to mediate viral entry (Rong and Bates, 1995). This domain of Tva is closely related to the ligand-binding domain of the low-density lipoprotein receptor (LDLR), which binds ligand *via* the interaction between acidic amino acids in the receptor and clustered basic residues in the ligand (Wilson et al., 1991). Analysis of the env sequence of ALV-A revealed a cluster of unique basic residues in hr2, suggesting a potential role of these residues in receptor recognition. Previous studies have shown that residues 210, 213, 223, 224, and 227 of the ALV-A SR-A isolate are important for effective infection (Rong et al., 1997). In addition, the alanine substitution of amino acids R213 or K227 reduced the receptor binding affinity by approximately 50%, while the alanine substitution of R210, R223, or R224 had no effect, suggesting that the effect of the basic residue mutations on envelope-mediated infection did not parallel the effect on receptor binding. However, our research showed an interesting result: the mutation R209Q, R222G, R223G, or K226I of the ALV-A RSA isolate (corresponding to residues R210, R223, R224, and K227 of SR-A) diminished the blocking effect against RCASBP (A)-EGFP (Figure 5F), suggesting a lower receptor binding affinity of these mutations, while the mutation R212K (corresponding to residue R213 of SR-A) had no effect (Figure 5D). This may be related to the basicity of L-lysine, as receptor binding was diminished significantly by alanine substitution but not by L-lysine substitution at R213 (Rong et al., 1997). In addition, although alanine substitution on R223 or R224 had no effect on receptor binding in a previous study, our research found that glycine substitution at the corresponding residue of ALV-A RSA could significantly reduce the blocking effect of viral entry (Figure 5F) and infectivity of DF-1 cells (Figure 5G), indicating that residues R222 and R223 of ALV-A RSA play an important role in receptor interaction. A similar result also appeared at residue Y141 (corresponding to residue Y142 of the ALV-A SR-A isolate) in hr1. Previous studies have revealed that the Y142N mutation of SR-A reduced the binding affinity of the env glycoproteins for quail but not chicken sTva-mIgG and the infectivity in cells expressing quail but not chicken Tva (Holmen et al., 2001). However, the chicken Tva receptor binding affinity and the infectivity in DF-1 cells were both significantly inhibited by mutating Y141 of ALV-A RSA to valine in our research (Figures 4B–D,I). Therefore, different amino acid mutations at the same site of SU may have different results on receptor interaction and virus invasion.

Due to the low fidelity in the reverse transcription process, ALV replicates with an extremely high mutation rate and exhibits huge genetic diversity, allowing the virus to quickly adapt to the external environment and develop resistance to the host immune response and antiviral drugs (Dong et al., 2017). Under external selection pressure, ALVs have the ability to change their cell invasion mechanism by evolving the structure of their Env proteins (Yin et al., 2019). ALV-A, in the presence of a competitive inhibitor, sTva-mIgG, evolved three variants (Y142N, W141G K261E, and W145R K261E) that may expand viral receptor usage while retaining wt levels of binding affinity for the chicken Tva receptor (Melder et al., 2003). In addition, the selected ALV-A variant had a six-amino acid deletion in residues 155–160 of hr1 in the presence of the subgroup A SU immune adhesin, expanding the use of Tvb and Tvc receptors and possibly other cell surface proteins for entry, while maintaining the ability to use the Tva receptor (Munguia and Federspiel, 2019). Our results showed that the substitutions W140 (corresponding to residue W141 of SR-A), Y141 (corresponding to residue Y142 of SR-A), s5 (aa153–155), and s6 (156–163) all had negative effects on receptor binding and viral infectivity (Figures 2, 4), suggesting a potential role of these residues in receptor recognition and viral invasion. In this study, utilizing a strategy of site-directed mutagenesis to substitute ALV-A SU with the corresponding sequence of ALV-E, a series of key amino acid positions that determine receptor interaction and virus invasion were systematically identified without excessively affecting the spatial conformation of the SU glycoprotein. However, this strategy cannot truly simulate the evolutionary mutation of ALV-A under the pressure of natural selection. Since ALV-A shares the Tva receptor with ALV-K, ALV-A SU could possibly evolve under selection pressure in the presence of ALV-K glycoprotein immune adhesins. Therefore, the evolutionary direction of ALV-A SU in the presence of ALV-K SU immune adhesins would be an interesting field to explore.

## Conclusion

The substitutions of residues s2 (aa134–139) to s12 (aa261–268) of ALV-A RSA with those of ALV-E ev-1 all reduced the blocking effect of viral entry, Tva binding affinity and the infectivity in DF-1 cells, and the substitutions of s3 (aa140–145), s5 (aa153–155), s8 (aa199–202), and s12 (aa261–268) had the most significant reduction. In addition, the substitutions of residues V138, W140, Y141, L142, S145, and L154 in hr1; residues V199, G200, Q202, R222, and R223 in hr2; or residue G262 in vr3 reduced the viral infectivity and the binding affinity of gp85 to Tva, similar to the results of the −139S, −151N, −155PWVNPF, −201NFD, Δ214–215, and −266S mutations. Our research has proven that the hr1 and hr2 domains of ALV-A contain principal receptor interaction determinants. However, in contrast to previous studies, here the vr3 domain was revealed to play a key role in the receptor binding affinity of ALV-A. This study will help to further clarify the infection mechanism of ALV-A.

## Data Availability Statement

The original contributions presented in the study are included in the article/Supplementary Material, further inquiries can be directed to the corresponding author.

## Author Contributions

JL, JC, ML, and WC designed the study, performed the experiments, and analyzed the data. CL, XD, XC, and MH helped to construct a series of RCASBP-based recombinant viruses and pCAGGS-based recombinant gp85 proteins. YG participated in the collection of literature and statistical analysis. All authors contributed to the article and approved the submitted version.

## Conflict of Interest

The authors declare that the research was conducted in the absence of any commercial or financial relationships that could be construed as a potential conflict of interest.

## Publisher’s Note

All claims expressed in this article are solely those of the authors and do not necessarily represent those of their affiliated organizations, or those of the publisher, the editors and the reviewers. Any product that may be evaluated in this article, or claim that may be made by its manufacturer, is not guaranteed or endorsed by the publisher.
