# Supplementary material for: Residues 140–142, 199–200, 222–223, and 262 in the Surface Glycoprotein of Subgroup A Avian Leukosis Virus Are the Key Sites Determining Tva Receptor Binding Affinity and Infectivity
Source: Front Microbiol. 2022 Apr 27;13:868377. doi: 10.3389/fmicb.2022.868377 (PMC9095613; doi:10.3389/fmicb.2022.868377)
Supplement: Supplementary file 1 [file Data_Sheet_1.docx]

Supplementary Material

# Supplementary Figures and Tables

## Supplementary Figures

**
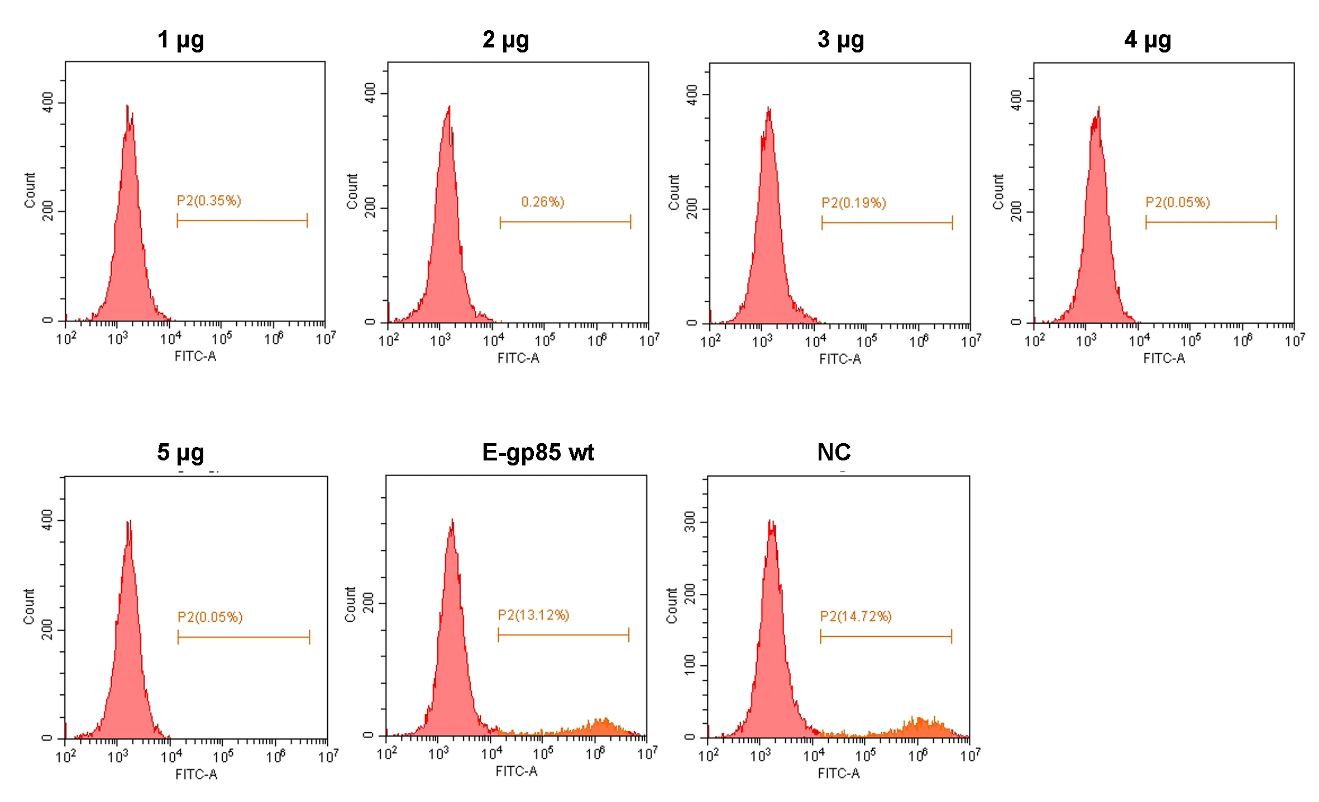
**

**Supplementary Figure S1.** Determination of optimal transfection amount of plasmid in blocking analysis. Using PolyJet DNA transfection reagent, 1, 2, 3, 4, and 5 µg of pCAGGS-RSA-s-gp85-flag plasmid, and 4 µg of pCAGGS-ev-1-s-gp85-flag plasmid was transfected into 293T cells in a 60 mm culture dish, respectively. At 8 h posttransfection, the cell supernatant was replaced by 3 mL of fresh DMEM with 1% FBS. DF-1 cells in 24-well were respectively incubated with the full volume of supernatant and subsequently infected with 0.1 MOI RCASBP (A)-EGFP supernatants at 48 h posttransfection. The percentage of GFP-positive cells was measured by FACS at 3 days postinfection.

**
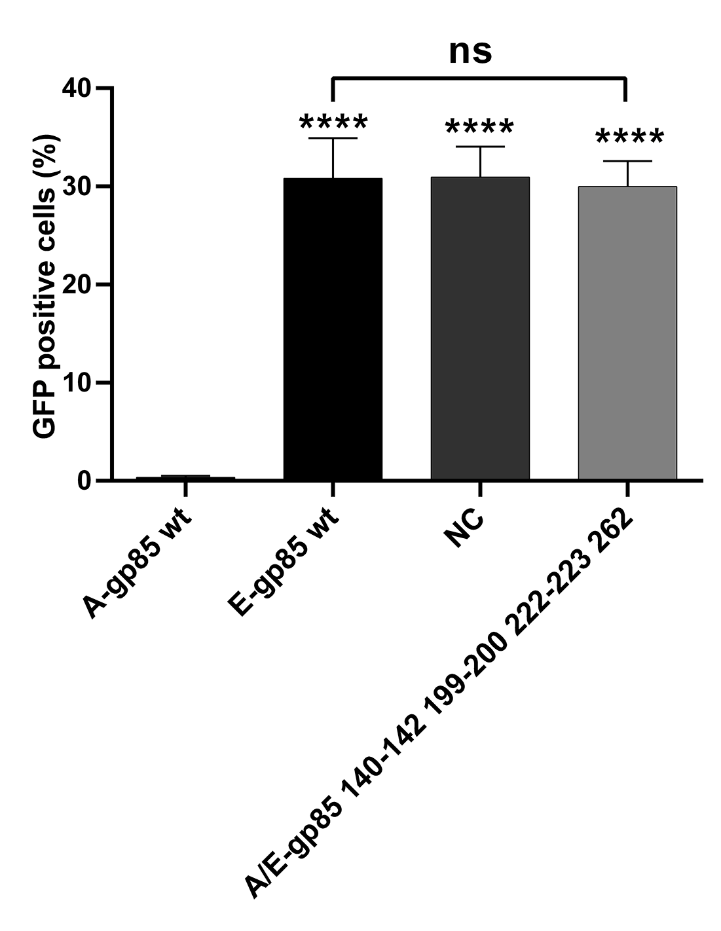
**

**Supplementary Figure S2.** Substitution of residues 140-142, 199-200, 222-223, and 262 of ALV-A with ALV-E exhibited no blocking effect on viral entry. DF-1 cells were incubated with ALV-A gp85 wt, ALV-E gp85 wt, DMEM, and chimeric gp85 proteins with substitution of residues 140-142, 199-200, 222-223, and 262 of ALV-A with ALV-E for 1 h at 4℃, respectively. After discarding the supernatant, DF-1 cells were subsequently infected with 0.1 MOI RCASBP (A)-EGFP supernatants for 2 h at 39℃. The percentage of GFP-positive cells was measured by FACS at 5 days postinfection for blocking analysis of the binding of chimeric gp85 protein to Tva.

## Supplementary Tables

**TABLE S1**. Primers for construction of recombinant RCASBP (A/E)-EGFP vectors

| **Primers^1^** | **Sequences 5' to 3'** | |
| --- | --- | --- |
| RCASBP-AE-KpnⅠ-F | | ACAGGGACACTGATAAGGTTATTTGggtaccCTCTCG^2^ |
| RCASBP-AE-StuⅠ-R | | CTTTCAGGCTGCCCACaggcctTTTGCAGCTTCTTATATTCC^2^ |
| RCASBP-AE-s1-F | | ACTCAGATTTCTGGTGTAACCGGGGGATGCGTAGGTT |
| RCASBP-AE-s1-R | | GTTACACCAGAAATCTGAGTAATATTAGTAATGTTAGGGAGAG |
| RCASBP-AE-s2-F | | GCCCCACACTCCAATCCTTGGTATCTAGGTTGGTCTAGAC |
| RCASBP-AE-s2-R | | CAAGGATTGGAGTGTGGGGCGAAACCTACGCATCCCCCGGTT |
| RCASBP-AE-s3-F | | AGTGGTGTCTACGGGTGGGGCAGACAGGAGGCCACGCGGTT |
| RCASBP-AE-s3-R | | GCCCCACCCGTAGACACCACTAGGAACCCCTTGTGGTCTGAAAC |
| RCASBP-AE-s4-F | | AGACAGGTTACACACAACTTTCTCCTTAGACACCCCTCTT |
| RCASBP-AE-s4-R | | AAGTTGTGTGTAACCTGTCTTCTAGACCAACCTAGATACC |
| RCASBP-AE-s5-F | | ATCGCCCCGTGGGTCAATCCTTTCCACCCCTCTTTCTCTAAATC |
| RCASBP-AE-s5-R | | GAAAGGATTGACCCACGGGGCGATGAGAAACCGCGTGGCCTCCT |
| RCASBP-AE-s6-F | | TTTAACAGCGCTTCTAACTCCACGGAACCGTTTACGGT |
| RCASBP-AE-s6-R | | GTTAGAAGCGCTGTTAAATCTAAGGAGAAACCGCGTGG |
| RCASBP-AE-s7-F | | GAAATATATAATTGCTCACACGTGGGGCGGCAGTACCGCT |
| RCASBP-AE-s7-R | | GTGTGAGCAATTATATATTTCCCAAAATCTGTAGCCATATGC |
| RCASBP-AE-s8-F | | AGATTTGATAATTTTGATATTTACCGCTGTGGTAATGCGCGC |
| RCASBP-AE-s8-R | | AATATCAAAATTATCAAATCTCTGTGAGCAGTTATACATGTT |
| RCASBP-AE-s9-F | | ACCTGTGGAGATGTGCAGACACCCCGCCCGGGTCTTCCTGAAAT |
| RCASBP-AE-s9-R | | TGTCTGCACATCTCCACAGGTGTACTGCCGCCCCACCTGTG |
| RCASBP-AE-s10-F | | GTCAAATCCCCCGAAAAACAGTGTACAAGGAGAGGAGG |
| RCASBP-AE-s10-R | | TGTTTTTCGGGGGATTTGACGCTGCGCGCATTACCACAGC |
| RCASBP-AE-S11-F | | GTGGGGGGAGGAGGTATATGGGTTAATCAATCACAGGAAAT |
| RCASBP-AE-s11-R | | CATATACCTCCTCCCCCCACACACTGGATTTCAGGAAGAC |
| RCASBP-AE-s12-F | | ACGACCACGATTCTCCCATCAGGGGCGTGGGTCGACAGCACACAAG |
| RCASBP-AE-s12-R | | CGCCCCTGATGGGAGAATCGTGGTCGTTTTTCCGCAACACCCACTG |
| RCASBP-AE-s2-V138N-F | | AATCCTTGGTATCTAGGTTGGTCTAGACAG |
| RCASBP-AE-s2-V138N-R | | ACCTAGATACCAAGGATTCCCTTGTGGTCTGAAACCT |
| RCASBP-AE-s3--139S-F | | GTTCCTAGTTGGTATCTAGGTTGGTCTAGACAG |
| RCASBP-AE-s3--139S-R | | ACCTAGATACCAACTAGGAACCCCTTGTGGTCTG |
| RCASBP-AE-s3-W140G-F | | GGTTATCTAGGTTGGTCTAGACAGGAGGCCACGCGGTT |
| RCASBP-AE-s3-W140G-R | | AGACCAACCTAGATAACCAGGAACCCCTTGTGGTCTG |
| RCASBP-AE-s3-Y141V-F | | TGGGTCCTAGGTTGGTCTAGACAGGAGGCCACGCGGTT |
| RCASBP-AE-s3-Y141V-R | | AGACCAACCTAGGACCCAAGGAACCCCTTGTGGTCTG |
| RCASBP-AE-s3-L142Y-F | | TGGTATTACGGTTGGTCTAGACAGGAGGCCACGCGGTT |
| RCASBP-AE-s3-L142Y-R | | AGACCAACCGTAATACCAAGGAACCCCTTGTGGTCTG |
| RCASBP-AE-s3-S145G-F | | TGGTATCTAGGTTGGGGCAGACAGGAGGCCACGCGGTT |
| RCASBP-AE-s3-S145G-R | | AGACCAACCTAGATAACCAGGAACCCCTTGTGGTCTG |
| RCASBP-AE-s4--151N-F | | AACTTTCTCCTTAGACACCCCTCTTTCT |
| RCASBP-AE-s4--151N-R | | GTGTCTAAGGAGAAAGTGCCGCGTGGCCTCCTGTCTAG |
| RCASBP-AE-s5-L154I-F | | GAGGCCACGCGGTTTCTCATCAGACACCCCTCTTTCTCT |
| RCASBP-AE-s5-L154I-R | | GATGAGAAACCGCGTGGCCTCCTGTCTAGACCAACCTAG |
| RCASBP-AE-s5-R155A-F | | TCCTTGCCCACCCCTCTTTCTCTAAATCCACGGAACCGT |
| RCASBP-AE-s5-R155A-R | | GAAAGAGGGGTGGGCAAGGAGAAACCGCGTGGCCTCCTGT |
| RCASBP-AE-s5--155PWVNPF-F | | CCGTGGGTCAATCCTTTCCACCCCTCTTTCTCTAAATC |
| RCASBP-AE-s5--155PWVNPF-R | | GAAAGGATTGACCCACGGTCTAAGGAGAAACCGCGTGG |
| RCASBP-AE-s8-V199R-F | | GCTCACAGAGAGGGCGGCAGTACCGCTGTGGTAATGC |
| RCASBP-AE-s8-V199R-R | | CTGCCGCCCTATCTGTGAGCAGTTATACATGTTCCAAAAT |
| RCASBP-AE-s8-G200F-F | | GCTCACAGGTGTTTCGGCAGTACCGCTGTGGTAATGC |
| RCASBP-AE-s8-G200F-R | | CTGCCGAAACACCTGTGAGCAGTTATACATGTTCCAAAAT |
| RCASBP-AE-s8-R201D-F | | GCTCACAGGTGGGGGATCAGTACCGCTGTGGTAATGC |
| RCASBP-AE-s8-R201D-R | | CTGATCCCCCACCTGTGAGCAGTTATACATGTTCCAAAAT |
| RCASBP-AE-s8--201NFD-F | | AATTTTGATCAGTACCGCTGTGGTAATGCGC |
| RCASBP-AE-s8--201NFD-R | | GCGGTACTGATCAAAATTCCGCCCCACCTGTGAGCAGT |
| RCASBP-AE-s8-Q202I-F | | GCTCACAGGTGGGGCGGATTTACCGCTGTGGTAATGC |
| RCASBP-AE-s8-Q202I-R | | AATCCGCCCCACCTGTGAGCAGTTATACATGTTCCAAAAT |
| RCASBP-AE-s10-∆214-215-F | | CGCACGCCTGAAATCCAGTGTACAAGGAGAGGAGG |
| RCASBP-AE-s10-∆214-215-R | | CTGGATTTCAGGCGGGCGGGGACTGCGCGCATTACCACAG |
| RCASBP-AE-s11-R222G-F | | GGGAGAGGAGGCAAATGGGTTAATCAAT |
| RCASBP-AE-s11-R222G-R | | CCATTTGCCTCCTCTCCCTGTACACTGGATTTCAGGAAG |
| RCASBP-AE-s11-R223G-F | | GGAGGAGGCAAATGGGTTAATCAATCAC |
| RCASBP-AE-s11-R223G-R | | AACCCATTTGCCTCCTCCCCTTGTACACTGGATTTCAG |
| RCASBP-AE-s12-A261T-F | | ACGGGCACGATTCTCCCGGGAAAGTGGGTCGACAGC |
| RCASBP-AE-s12-A261T-R | | CCGGGAGAATCGTGCCCGTTTTTCCGCAACACCCACT |
| RCASBP-AE-s12-G262T-F | | GCAACCACGATTCTCCCGGGAAAGTGGGTCGACAGCAC |
| RCASBP-AE-s12-G262T-R | | TCCCGGGAGAATCGTGGTTGCTTTTCCGCAACACCCACT |
| RCASBP-AE-s12--266S-F | | TTCTCCCGTCAGGAAAGTGGGTCGACAGCACAC |
| RCASBP-AE-s12--266S-F | | CCACTTTCCTGACGGGAGAATCGTGCCTGCTT |
| RCASBP-AE-s12-K268A-F | | TTCTCCCGGGAGCGTGGGTCGACAGCACACAAGGTAGT |
| RCASBP-AE-s12-K268A-R | | GACCCACGCTCCCGGGAGAATCGTGCCTGCTTTTCCGC |

^1^F, forward primer; R, reverse primer. ^2^The lowercase letters were the sites for restriction digest.

**TABLE S2**. Primers for construction of plasmids expressing chimeric gp85 proteins

| **Primers^1^** | **Sequences 5' to 3'** | |
| --- | --- | --- |
| pCAGGS-AE-gp85-flag-EcoRⅠ-F | | CTCATCATTTTGGCAAAgaattcATGGATAGCAAGGGCAGCAG^2^ |
| pCAGGS-AE-gp85-flag-BglⅡ-R | | TGGCAGAGGGAAAAagatctTTATTTGTCATCGTCATCT^2^ |
| pCAGGS-AE-gp85-s1-F | | ACTCAGATCAGCGGAGTAACCGGCGGCTGCGTGGGCTT |
| pCAGGS-AE-gp85-s1-R | | TACTCCGCTGATCTGAGTGATGTTGGTGATGTTGGGC |
| pCAGGS-AE-gp85-s2-F | | CTTTGCCCCTCACTCCAATCCTTGGTATCTGGGATGGTCT |
| pCAGGS-AE-gp85-s2-R | | ATTGGAGTGAGGGGCAAAGCCCACGCAGCCGCCG |
| pCAGGS-AE-gp85-s3-F | | AGTGGTGTCTATGGATGGGGCAGACAAGAGGCCACAAGATTTC |
| pCAGGS-AE-gp85-s3-R | | GCCCCATCCATAGACACCACTAGGCACGCCTTGAGGTCTAA |
| pCAGGS-AE-gp85-s4-F | | AGACAGGTTACACACAACTTTCTGCTGAGGCACCCCAG |
| pCAGGS-AE-gp85-s4-R | | AAGTTGTGTGTAACCTGTCTTCTAGACCATCCCAGATACCAAG |
| pCAGGS-AE-gp85-s5-F | | ATCGCCCCTTGGGTCAATCCTTTCCACCCCAGCTTTTCCAAAAG |
| pCAGGS-AE-gp85-s5-R | | GAAAGGATTGACCCAAGGGGCGATCAGAAATCTTGTGGCCTCTT |
| pCAGGS-AE-gp85-s6-F | | TTCAACAGCGCCTCTAACAGCACCGAGCCTTTCACCGT |
| pCAGGS-AE-gp85-s6-R | | GTTAGAGGCGCTGTTGAACCTCAGCAGAAATCTTGTGG |
| pCAGGS-AE-gp85-s7-F | | GAAATATACAACTGCAGCCACGTGGGAAGACAGTATAGATG |
| pCAGGS-AE-gp85-s7-R | | GTGGCTGCAGTTGTATATTTCCCAGAACCTATAGCCGTAGG |
| pCAGGS-AE-gp85-s8-F | | AGATTTGATAATTTTGATATTTATAGATGTGGCAATGCCAG |
| pCAGGS-AE-gp85-s8-R | | AATATCAAAATTATCAAATCTTTGGCTGCAGTTGTACATGT |
| pCAGGS-AE-gp85-s9-F | | ACCTGTGGCGATGTGCAGACACCTAGACCCGGACTGCCC |
| pCAGGS-AE-gp85-s9-R | | TGTCTGCACATCGCCACAGGTATACTGTCTTCCCACTTGG |
| pCAGGS-AE-gp85-s10-F | | GTCAAATCCCCCGAGAAACAGTGCACAAGAAGGG |
| pCAGGS-AE-gp85-s10-R | | TTTCTCGGGGGATTTGACGCTTCTGGCATTGCCACATC |
| pCAGGS-AE-gp85-s11-F | | GTGGGGGGAGGCGGCATATGGGTCAACCAGAGCCAAGAG |
| pCAGGS-AE-gp85-s11-R | | TATGCCGCCTCCCCCCACGCACTGGATCTCGGGCAGTC |
| pCAGGS-AE-gp85-s12-F | | ACGACCACAATTCTCCCCTCAGGAGCGTGGGTGGATAGCAC |
| pCAGGS-AE-gp85-s12-R | | AGGGGAGAATTGTGGTCGTCTTGCCGCAGCAGCCGCT |
| pCAGGS-AE-s2-R134A-F | | GCCCCTCAAGGCGTGCCTTGGTATCTGGGAT |
| pCAGGS-AE-s2-R134A-R | | AGGCACGCCTTGAGGGGCAAAGCCCACGCAGCCG |
| pCAGGS-AE-s2-Q136H-F | | AGACCTCACGGCGTGCCTTGGTATCTGGGAT |
| pCAGGS-AE-s2-Q136H-R | | AGGCACGCCGTGAGGTCTAAAGCCCACGCAGCCG |
| pCAGGS-AE-s2-G137S-F | | AGACCTCAATCCGTGCCTTGGTATCTGGGAT |
| pCAGGS-AE-s2-G137S-R | | AGGCACGGATTGAGGTCTAAAGCCCACGCAGCCG |
| pCAGGS-AE-s2-V138N-F | | AGACCTCAAGGCAATCCTTGGTATCTGGGAT |
| pCAGGS-AE-s2-V138N-R | | AGGATTGCCTTGAGGTCTAAAGCCCACGCAGCCG |
| pCAGGS-AE-s3--139S-F | | TCAAGGCGTGCCTAGCTGGTATCTGGGATGGTCT |
| pCAGGS-AE-s3--139S-R | | ACCAGCTAGGCACGCCTTGAGGTCTAAAGC |
| pCAGGS-AE-s3-W140G-F | | GGTTATCTGGGATGGTCTAGACAAGAGGCCACAAGATTTC |
| pCAGGS-AE-s3-W140G-R | | AGACCATCCCAGATAGCCAGGCACGCCTTGAGGTCTAA |
| pCAGGS-AE-s3-Y141V-F | | TGGGTGCTGGGATGGTCTAGACAAGAGGCCACAAGATTTC |
| pCAGGS-AE-s3-Y141V-R | | AGACCATCCCAGCACCCAAGGCACGCCTTGAGGTCTAA |
| pCAGGS-AE-s3-L142Y-F | | TGGTATTACGGATGGTCTAGACAAGAGGCCACAAGATTTC |
| pCAGGS-AE-s3-L142Y-R | | AGACCATCCGTAATACCAAGGCACGCCTTGAGGTCTAA |
| pCAGGS-AE-s3-S145G-F | | TGGTATCTGGGATGGGGCAGACAAGAGGCCACAAGATTTC |
| pCAGGS-AE-s3-S145G-R | | GCCCCATCCCAGATACCAAGGCACGCCTTGAGGTCTAA |
| pCAGGS-AE-s4-Q147R-F | | TGGTCTAGAAGAGAGGCCACAAGATTTCTG |
| pCAGGS-AE-s4-Q147R-R | | GTGGCCTCTCTTCTAGACCATCCCAGATAC |
| pCAGGS-AE-s4-E148Q-F | | AGACAACAGGCCACAAGATTTCTGCTGAGG |
| pCAGGS-AE-s4-E148Q-R | | AATCTTGTGGCCTGTTGTCTAGACCATCC |
| pCAGGS-AE-s4-A149V-F | | CAAGAGGTTACAAGATTTCTGCTGAGGCAC |
| pCAGGS-AE-s4-A149V-R | | AGAAATCTTGTAACCTCTTGTCTAGACCAT |
| pCAGGS-AE-s4-R151H-F | | GAGGCCCACAGATTTCTGCTGAGGCACC |
| pCAGGS-AE-s4-R151H-R | | AGCAGAAATCTGTGGGCCTCTTGTCTAGACCAT |
| pCAGGS-AE-s4--151N-F | | CACAAGAAACTTTCTGCTGAGGCACCCCAG |
| pCAGGS-AE-s4--151N-R | | CAGCAGAAAGTTTCTTGTGGCCTCTTGTCTAG |
| pCAGGS-AE-s5-L154I-F | | GCCACAAGATTTCTGATCAGGCACCCCAGCTTTTCCAAAAG |
| pCAGGS-AE-s5-L154I-R | | CCTGATCAGAAATCTTGTGGCCTCTTGTCTAGACCATCC |
| pCAGGS-AE-s5-R155A-F | | GCCACAAGATTTCTGCTGGGCCACCCCAGCTTTTCCAAAAG |
| pCAGGS-AE-s5-R155A-R | | GCCCAGCAGAAATCTTGTGGCCTCTTGTCTAGACCATCC |
| pCAGGS-AE-s5--155PWVNPF-F | | CCTTGGGTGAACCCTTTCCACCCCAGCTTTTCCAAAAG |
| pCAGGS-AE-s5--155PWVNPF-R | | GAAAGGGTTCACCCAAGGCCTCAGCAGAAATCTTGTGG |
| pCAGGS-AE-s6-H156F-F | | TTCCCCAGCTTTTCCAAAAGCACCGAGCCTT |
| pCAGGS-AE-s6-H156F-R | | TTTGGAAAAGCTGGGGAACCTCAGCAGAAATCT |
| pCAGGS-AE-s6-P157N-F | | CACAACAGCTTTTCCAAAAGCACCGAGCCTT |
| pCAGGS-AE-s6-P157N-R | | TTTGGAAAAGCTGTTGTGCCTCAGCAGAAATCT |
| pCAGGS-AE-s6-F159A-F | | CACCCCAGCGCTTCCAAAAGCACCGAGCCTT |
| pCAGGS-AE-s6-F159A-R | | TTTGGAAGCGCTGGGGTGCCTCAGCAGAAATCT |
| pCAGGS-AE-s6-H161N-F | | CACCCCAGCTTTTCCAACAGCACCGAGCCTT |
| pCAGGS-AE-s6-H161N-R | | GTTGGAAAAGCTGGGGTGCCTCAGCAGAAAT |
| pCAGGS-AE-s7-N192E-F | | GAGATGTACAACTGCAGCCAAGTGGGAAGAC |
| pCAGGS-AE-s7-N192E-R | | GCTGCAGTTGTACATCTCCCAGAACCTATAGCCGTAG |
| pCAGGS-AE-s7-M193I-F | | AACATCTACAACTGCAGCCAAGTGGGAAGAC |
| pCAGGS-AE-s7-M193I-R | | GCTGCAGTTGTAGATGTTCCAGAACCTATAGC |
| pCAGGS-AE-s7-Q198H-F | | AGCCACGTGGGAAGACAGTATAGATGT |
| pCAGGS-AE-s7-Q198H-R | | CTGTCTTCCCACGTGGCTGCAGTTGTACAT |
| pCAGGS-AE-s8-V199R-F | | AGAGGAAGACAGTATAGATGTGGCAATGCCAGAAGCCCT |
| pCAGGS-AE-s8-V199R-R | | CACATCTATACTGTCTTCCTCTTTGGCTGCAGTTGTACATGT |
| pCAGGS-AE-s8-G200F-F | | GTGTTCAGACAGTATAGATGTGGCAATGCCAGAAGCCCT |
| pCAGGS-AE-s8-G200F-R | | CACATCTATACTGTCTGAACACTTGGCTGCAGTTGTACATGT |
| pCAGGS-AE-s8-R201D-F | | GTGGGAGATCAGTATAGATGTGGCAATGCCAGAAGCCCT |
| pCAGGS-AE-s8-R201D-R | | CACATCTATACTGATCTCCCACTTGGCTGCAGTTGTACATGT |
| pCAGGS-AE-s8--201NFD-F | | AACTTCGATCAGTATAGATGTGGCAATGCCAGAAG |
| pCAGGS-AE-s8--201NFD-R | | ACATCTATACTGATCGAAGTTTCTTCCCACTTGGCTGCAGT |
| pCAGGS-AE-s8-Q202I-F | | GTGGGAAGAATCTATAGATGTGGCAATGCCAGAAGCCCT |
| pCAGGS-AE-s8-Q202I-R | | CACATCTATAGATTCTTCCCACTTGGCTGCAGTTGTACATGT |
| pCAGGS-AE-s9-R204T-F | | ACCTGTGGCAATGCCAGAAGCCCTAGAC |
| pCAGGS-AE-s9-R204T-R | | TCTGGCATTGCCACAGGTATACTGTCTTCCCACTTG |
| pCAGGS-AE-s9-N207D-F | | TGGCGACGCCAGAAGCCCTAGACCCGGACT |
| pCAGGS-AE-s9-N207D-R | | AGGGCTTCTGGCGTCGCCACATCTATACTGTCTTC |
| pCAGGS-AE-s9-A208V-F | | GCAATGTGAGAAGCCCTAGACCCGGACTG |
| pCAGGS-AE-s9-A208V-R | | TAGGGCTTCTCACATTGCCACATCTATACTGTC |
| pCAGGS-AE-s9-R209Q-F | | ATGCCCAAAGCCCTAGACCCGGACTGC |
| pCAGGS-AE-s9-R209Q-R | | GTCTAGGGCTTTGGGCATTGCCACATCTATACT |
| pCAGGS-AE-s9-S210T-F | | AGAACACCTAGACCCGGACTGCCCGAG |
| pCAGGS-AE-s9-S210T-R | | TCCGGGTCTAGGTGTTCTGGCATTGCCACATCTAT |
| pCAGGS-AE-s10-P211V-F | | AGAAGCGTCAGACCCGGACTGCCCGAGAT |
| pCAGGS-AE-s10-P211V-R | | TCCGGGTCTGACGCTTCTGGCATTGCCAC |
| pCAGGS-AE-s10-R212K-F | | AGCCCTAAGCCCGGACTGCCCGAGATCCAG |
| pCAGGS-AE-s10-R212K-R | | GCAGTCCGGGCTTAGGGCTTCTGGCATTGC |
| pCAGGS-AE-s10-P213S-F | | CCTAGATCCGGACTGCCCGAGATCCAGT |
| pCAGGS-AE-s10-P213S-R | | GCAGTCCGGATCTAGGGCTTCTGGCAT |
| pCAGGS-AE-s10-∆214-215-F | | GCCCTAGACCCCCCGAGATCCAGTGCACAAG |
| pCAGGS-AE-s10-∆214-215-R | | GATCTCGGGGGGTCTAGGGCTTCTGGCATT |
| pCAGGS-AE-s10-I218K-F | | TGCCCGAGAAGCAGTGCACAAGAAGGGGC |
| pCAGGS-AE-s10-I218K-R | | TGTGCACTGCTTCTCGGGCAGTCCGGGTCT |
| pCAGGS-AE-s11-T221V-F | | GTGAGAAGGGGCGGCAAGTGGGTCAAC |
| pCAGGS-AE-s11-T221V-R | | CTTGCCGCCCCTTCTCACGCACTGGATCTCGGGCAGT |
| pCAGGS-AE-s11-R222G-F | | GGCAGGGGCGGCAAGTGGGTCAAC |
| pCAGGS-AE-s11-R222G-R | | ACTTGCCGCCCCTGCCTGTGCACTGGATCTCGGGC |
| pCAGGS-AE-s11-R223G-F | | GGCGGCGGCAAGTGGGTCAACCAGAG |
| pCAGGS-AE-s11-R223G-R | | TGACCCACTTGCCGCCGCCTCTTGTGCACTGGATCT |
| pCAGGS-AE-s11-K226I-F | | ATCTGGGTCAACCAGAGCCAAGAGAT |
| pCAGGS-AE-s11-K226I-R | | GCTCTGGTTGACCCAGATGCCGCCCCTTCTTGTGC |
| pCAGGS-AE-s12-A261T-F | | ACAGGAACAATTCTCCCCGGAAAGTGGGTGGATAGCACC |
| pCAGGS-AE-s12-A261T-R | | CGGGGAGAATTGTTCCTGTCTTGCCGCAGCAGCCGCT |
| pCAGGS-AE-s12-G262T-F | | GCTACCACAATTCTCCCCGGAAAGTGGGTGGATAGCACC |
| pCAGGS-AE-s12-G262T-R | | CGGGGAGAATTGTGGTAGCCTTGCCGCAGCAGCCGCT |
| pCAGGS-AE-s12--266S-F | | GAACAATTCTCCCCTCCGGAAAGTGGGTGGATAGCAC |
| pCAGGS-AE-s12--266S-R | | CCGGAGGGGAGAATTGTTCCAGCCTTGCCGCAGC |
| pCAGGS-AE-s12-K268A-F | | GAACAATTCTCCCCGGAGCTTGGGTGGATAGCACCCAAGGCT |
| pCAGGS-AE-s12-K268A-R | | AGCTCCGGGGAGAATTGTTCCAGCCTTGCCGCAGC |

^1^F, forward primer; R, reverse primer. ^2^The lowercase letters were the sites for restriction digest.
