# Supplementary figures and images for: Residues 140–142, 199–200, 222–223, and 262 in the Surface Glycoprotein of Subgroup A Avian Leukosis Virus Are the Key Sites Determining Tva Receptor Binding Affinity and Infectivity
Source: Front Microbiol. 2022 Apr 27;13:868377. doi: 10.3389/fmicb.2022.868377 (PMC9095613; doi:10.3389/fmicb.2022.868377)

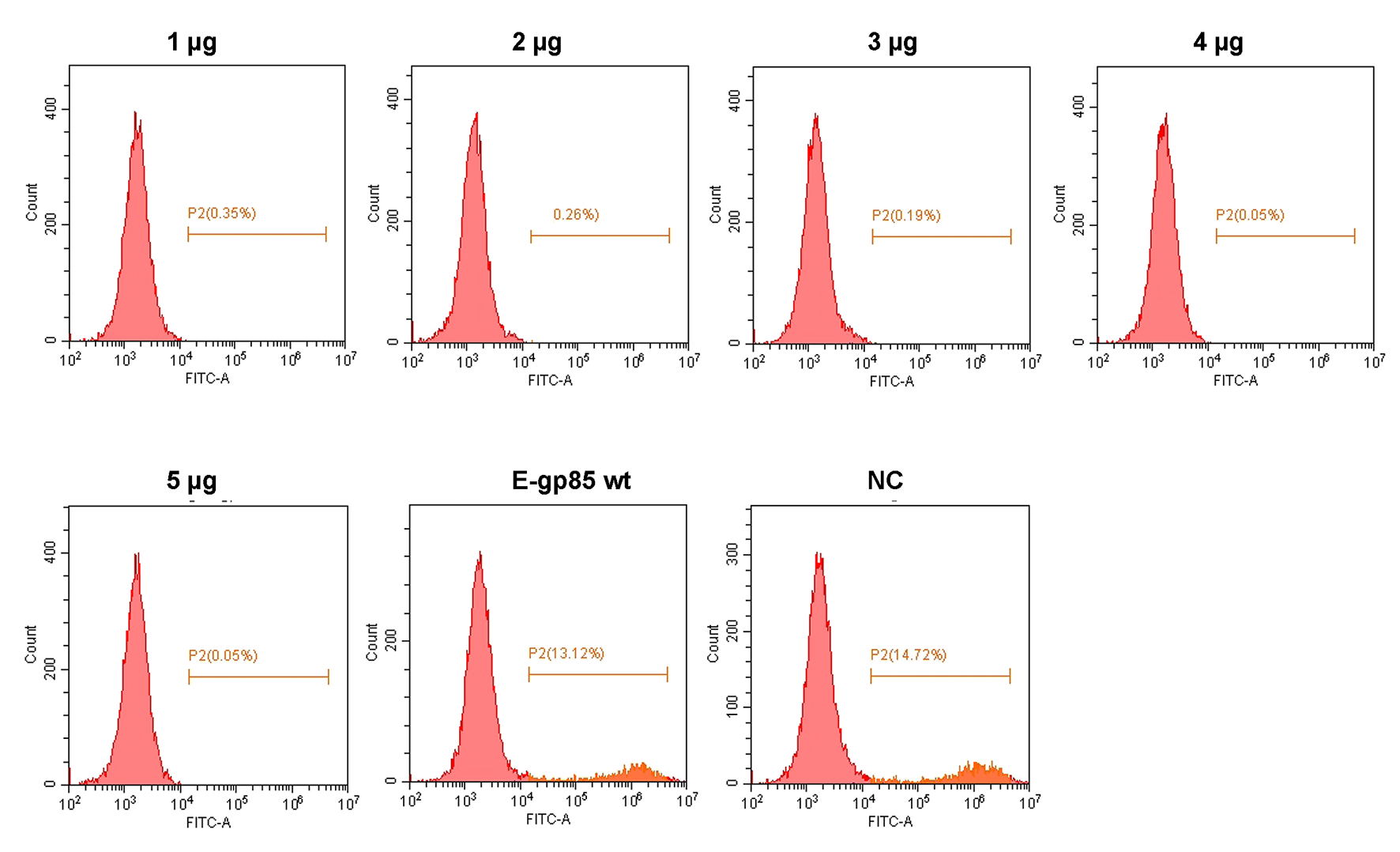

Supplement: Supplementary file 2 [file Image_1.tif]

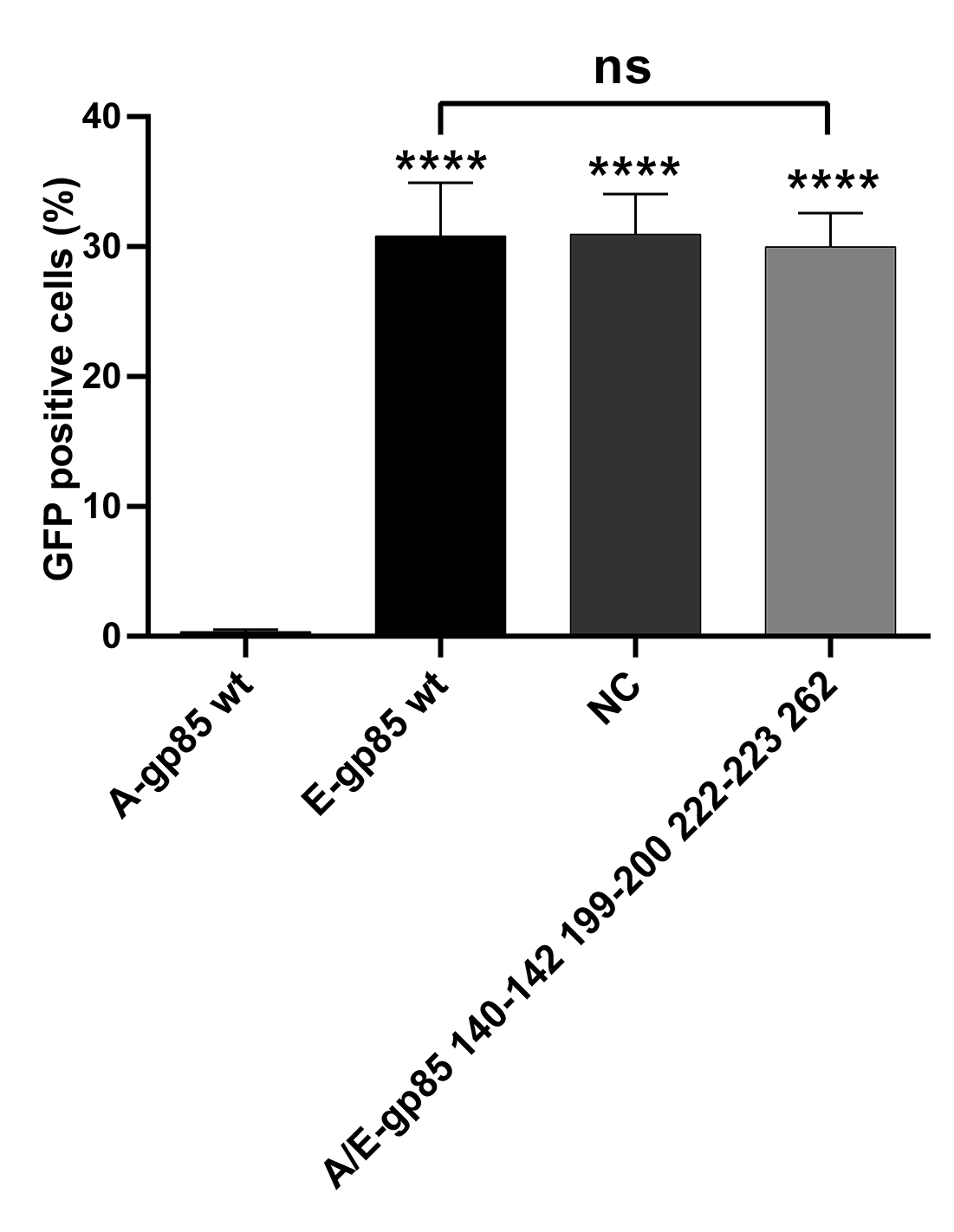

Supplement: Supplementary file 3 [file Image_2.tif]
